# Co-designing MOOCs with CoDe-Graph

**DOI:** 10.1007/s41686-022-00064-2

**Published:** 2022-02-28

**Authors:** Allison L. G. Kolling, Armin Weinberger, Helmut Niegemann

**Affiliations:** Saarland University, Saarbrücken, Germany

**Keywords:** Co-design, MOOCs, Graphical modeling languages, Shared artifacts, Design process

## Abstract

As MOOCs have become a standard format of online learning, it is increasingly important to design courses that fit the needs and contexts of the targeted learners. One way to do so is by actively designing with the subject experts, instructors, and other stakeholders. Within the context of designing MOOCs for disadvantaged groups in Southeast Asia, we explore the three-phase process of co-design. We present a graphical modeling language, CoDe-Graph, which can be used to facilitate the co-design process. We examine how diverse groups of experts provide feedback on design elements and create a common understanding using shared artifacts. Four case studies illustrate how the tool can be used by co-design teams to create and visualize custom MOOC designs.

## MOOC Design: Does One Size Fit All?

Online learning has become common across educational institutions around the world. MOOCs (Massive Open Online Courses) are available to anyone with an internet connection and indeed, by 2020 over one hundred eighty million people have enrolled in a MOOC (Shah, 2020). While this has been raising hopes for democratizing education, i.e., making higher education available to many around the world who would otherwise be excluded (Dillahunt et al., 2014), the dominant format of the xMOOC, the extension of regular lectures entailing highly self-directed learning with video lectures and quizzes, raised complaints of students about not having sufficient access to instructors or peers (Baggaley, 2013). These courses tend to inadvertently favor those with experience in formal higher educational institutions over disadvantaged populations (Glass et al., 2016; Liyanagunawardena et al., 2013). The alternative cMOOC design, which is completely open and self-driven, also poses challenges for non-traditional learners who tend to benefit from structure and high levels of guidance (Kop et al., 2011). Democratizing education may need to start with customizing MOOC designs that take the culture, educational levels, motivation, lifestyle, and available infrastructure of the target audience into account. Creating such customized designs is extremely difficult for instructional designers outside of the respective target community, and despite best efforts, the resulting programs and tools are often not adopted by local practitioners in the long term, but rather are abandoned after testing (Anderson & Shattuck, 2012).

Customizing courses to the needs of very specific populations necessitates the involvement of those populations in the design process itself. One way to do this is to use a method of participatory design or co-design, which can be defined as the practice of involving multiple stakeholder groups in the design and decision-making process (Roschelle et al., 2006). This has become more common in education in recent years as it tends to increase acceptance and engagement among educators (Rodríguez-Triana et al., 2020). However, co-design is still relatively new and there are significant challenges to the approach. Among these challenges is facilitating co-design in such a way as to promote the development of a shared vision and mutual understanding among the varied experts (Penuel et al., 2007).

This article aims to investigate the facilitation of co-design sessions of MOOCs for disadvantaged groups. First, we will lay out the practice of co-design, its role in education, and the challenges of co-design. Next, we will introduce a process model that can be used to structure co-design sessions. In doing so, we will also explore the role of shared artifacts in co-design and present a graphical language for co-designing MOOCs (CoDe-Graph), which visually represents design decisions. Furthermore, we will illustrate case studies of MOOCs emerging from such a systematic co-design approach using CoDe-Graph. Finally, we will discuss the implication of these case studies on future co-design research and practices.

## Co-design in Education

### Definition and Use in Educational Contexts

Co-design in education generally refers to the collaboration between researchers and teachers to produce learning objects and environments (Barbera et al., 2017; Cober et al., 2015). The co-design process differs from other methods of design in that it operates “bottom-up” with teachers being active participants in the design process, who provide critical insight into daily work practices and the existing learning culture.

The involvement of educators and other stakeholders in the co-design process does not demand equal expertise and participation from participants. Consequently, co-design builds on participants having different roles and ultimately diverse responsibilities (Penuel et al., 2007). Co-design is then defined as:

a highly facilitated, team-based process in which teachers, researchers, and developers work together in defined roles to design an educational innovation, realize the design in one or more prototypes, and evaluate each prototype’s significance for addressing a concrete educational need (Roschelle et al., 2006).

Co-design is used in a wide variety of contexts in education and may be well suited for use with e-learning environments and tools, as technicians may not have the content and pedagogical knowledge needed to envision which tools are appropriate and teachers rarely have the technological skills needed to create e-learning tools themselves (Cober et al., 2015). In the past, technology was perceived to allow introducing advanced and complex pedagogies in schools, such as problem-oriented learning, anchored instruction, or design-based learning. Researchers could design for and analyze how learners develop applicable knowledge and skills with hopes of scaling up such approaches through technology and changing the educational culture towards active and social forms of learning. More often than not, however, teachers used the technology-enhanced learning environment as a quarry for multi-media material to integrate into their traditional teaching after the researchers left (Anderson & Shattuck, 2012). This may be a problem of cultural changes being difficult and protracted when they involve a conceptual change of teachers, but also incompatibility of the respective environments with practical issues concerning teachers’ technological prowess and equipment as well as fitting with the curriculum in regard to time and contents (Kollar & Fischer, 2008).

When developing educational technologies, co-design has several advantages and is generally beneficial for both researchers and instructors. Researchers gain insight into the educational contexts in which their tools will be deployed (Cober et al., 2015). This in turn helps to ensure that the environments or tools being designed to meet the needs and goals of the educator and increases the likelihood of continuous implementation and use of the new technologies. Co-design essentials aim to create sustainable learning environments that continue to be used after researchers leave the field. In the case of creating digital formative assessment tools, teachers, some of whom had originally been reluctant to participate, eventually took ownership of the resulting software and become advocates for its expanded implementation (Roschelle et al., 2006). Similarly, in a comparative study, where teachers were assigned one of three roles in the design of early literacy tools, executor, re-designer, or co-designer, those cases in which teachers were co-designers implemented more technology-enhanced tools and had greater acceptance and implementation (Cviko et al., 2014).

In addition, co-design led to an increase in teachers learning through self-reflection and sharing perspectives on how to teach and learn (Matuk et al., 2016; Penuel et al., 2007; Roschelle et al., 2006). While experiencing a relative increase in use, co-design processes and approaches remain under-specified in education.

### Challenges of Co-design

Despite its promise, problems emerge within the co-design process. Bringing diverse groups of experts together at one table does not suffice for a productive exchange of expertise and perspectives (Durall et al., 2019). For instance, computer scientists may lack the domain language and specific examples of how tools, methods, and learning activities can be used within a learning context. Similarly, instructors may have difficulties expressing their pedagogical ideas in a non-ambiguous, highly formalized way that computer scientists can convert into online learning environments. Both groups may struggle to communicate with the researchers who are focused on bringing theories into practice. These different perspectives and expectations can make the co-design process frustrating for participants (Sanders & Stapper, 2008; Penuel et al., 2007).

In fact, facilitating and supporting communication about design ideas and learning activities between different stakeholders, in particular between researchers, programmers, and instructors, has become a significant challenge in co-design. Co-design processes benefit greatly from facilitation, which encourages participants to utilize the unique skills and experience each person contributes to the group (Durall et al., 2019). As the responsibility for the design is centralized within co-design (Penuel et al., 2007), the team leads must find a way to scaffold participation to maximize the advantages of co-design (Voogt et al., 2016). As a result, there have been numerous attempts to systemize how participants interact with each other and the materials. One approach has been the use of design games where each participant fulfills a specified role while moving through the design process (Brandt, 2006). In such games, participants are both encouraged to be playful and creative and to stay within the tightly established boundaries of the game (Vaajakallio & Mattelmäki, 2014). In contrast, others have more loosely defined informal roles such as providing a needs analysis or feedback on prototypes giving greater flexibility and encouraging a relaxed atmosphere which builds trust (Cober et al., 2015). In any case, providing support for stakeholders, particularly educators, to work efficiently and effectively with researchers is needed in co-design sessions (Matuk et al., 2016).

### Design Methods—Iterative Design Through SAM

Co-design is only one aspect of the larger design process. Independent of the decision to include stakeholders is the decision of how to structure the design process itself. Each research team has approached co-design differently, but a general trajectory can be identified in several cases. Roschelle et al. (2006) identify three stages in co-design: collecting requirements, rapid prototyping, and software solidification. Additionally, feedback rounds have been included as possible intermediate steps in the co-design process to improve the prototype (Cober et al., 2015).

For years, sequential design approaches such as ADDIE have been the dominant design approach in learning. This “waterfall” model allows designers to proceed through design steps quickly and efficiently moving towards the final product. However logical this model is, it does come with limitations, often a delay in a single step can delay the entire process and changes are difficult to implement once a step has been completed. For this reason, there has been an introduction of more flexible design approaches, which work through iterations. One such method is the Successive Approximation Model or SAM (Allen, 2018).

SAM consists of repeating steps to get continuously closer to the perfect design. This formative approach allows participants to continuously evaluate and adapt their approach until they are satisfied with the results. SAM consists of two or in larger projects three phases. In both models, the first stage is information gathering. In SAM, information gathering starts by collecting information from key players and ends with an event referred to as the “Savvy Start,” during which participants present their initial design ideas (Allen, 2018, p. 44). In co-design research, this stage is particularly important as it reduces ambiguity and helps participants understand the goals, perspectives, and expectations of their team members. Within the co-design workshops, various methods and tools such as analyzing the use of physical artifacts, user stories, and reacting to scenarios can be used to facilitate the pre-design requirement stage (Matuk et al., 2016).

SAM then transitions to the design stage, which consists of designing, building, and reviewing alternatives. Typically, the design tasks are repeated three times to ensure that there is ample chance for better alternatives to emerge and to increase confidence in the selected design. In the larger model, iterative rounds of implementation and review are included in a third stage (Allen, 2018). This approach has served as the basis for formative design studies in education in recent years. For instance, Schmidt and colleagues (2020) used SAM as a basis for their own design model when creating a training course for caregivers of youth with traumatic brain injuries. In this case, the model has been adapted to include consultations with subject experts, potential users, and technicians as well as a literature review. Three design rounds and three implementation rounds were each accompanied by reflection and evaluation phases. Improvements in each design cycle led to high levels of user satisfaction.

SAM may be particularly well suited for co-design projects as it allows for, and even encourages, the participation of various stakeholders throughout the process. In addition, it is a lean and flexible approach that is accessible to non-experts. Additionally, it allows for formative design with constant evaluations and improvements at each step of the process. In the following sections, we will propose a process model for co-design that is based on the SAM model but fine-tuned to supporting groups of mixed expertise and creating complex course designs in short amounts of time. This model is also sequential, but unlike SAM, the process is not repeated in full, but it relies on micro-iterations of the design and evaluation processes to reach a product that meets all stakeholder’s needs and expectations. The three-phase model allows for scaffolding the design process using scripted interviews and Educational Modelling Languages.

## Towards a Process Model for Co-design

The process model of a co-design session is divided into three phases: establishing context, design, and presentation. Each phase will be further elaborated on in this paper. In addition to the division by phase, the model is also split by role: facilitators and stakeholders.

The facilitating team is primarily responsible for the organization and moderation of the session and the stakeholders for the content creation and technical realization. While defining the roles of educators and other stakeholders is an extensive field of study within co-design, here we will restrict ourselves to identifying macro-roles and responsibilities that the groups have within each phase. These will be further detailed in the relevant sections below.

### Phase One: Establishing Context

Similar to the information gathering stage in SAM, during the first phase of establishing context, facilitators elicit information from the local stakeholders about the context of the course. The objective of this stage is to create a shared understanding of aspects such as content, resources, and challenges of the course. In this phase, it is up to the local stakeholders to inform the facilitators of the situation “on the ground,” and to set realistic expectations for what is and is not possible in their contexts. Having multidisciplinary teams involved in this process allowed for the exchange of information between not only the facilitators and local stakeholders, but also between stakeholder groups. All stakeholders should have the opportunity to share their expertise and learn from one another. Technologists present the available technology, its functions, and limitations; teachers provide course goals and content; and community members offer information about the target users and cultural context. The facilitators’ team leads this discussion. One option for achieving this is using a semi-structured interview, deploying questions such as “What is the average age and education level of a participant?” “How long should a learning session last?” or “Does the course end with a formal certification? If so, what will participants have to accomplish in order to receive certification?” This set of guiding questions then becomes the basis of collective understanding and creates a baseline from which to operate. It also gives multiple opportunities to check for understanding and correct miscommunications as all participants have equal access to the document and can edit it in real time during the discussion.

### Phase Two: Design

Once the first phase has been concluded, the group moves to the second stage, design. In this iterative phase, ideas should be generated and auditioned. Individual team members are encouraged to make proposals, adapt, or expand on ideas and ask questions. The stakeholder group then discusses the suggestions and provides feedback. While both the facilitators and stakeholders actively participate in this phase, the facilitators generally restrict themselves to asking guiding or clarifying questions. This is represented as a cycle since multiple suggestions and feedback rounds occur before arriving at a consensus within a small group. During each cycle, every member has multiple opportunities to make suggestions, add to the ideas of others, and express criticism. A common trend among both high- or low-structured approaches to co-design is the use of physical artifacts to support a shared understanding of the design (Barbera et al., 2017). Co-design games include props such as playing cards and game pieces (Brandt, 2006) and case studies as well as rapid prototyping are used to anchor discussions and lead towards shared understanding (Matuk et al., 2016). Digital and analog sketches also have been used to help focus participants on a common vision (Cviko et al., 2014; McKenney & Mor, 2015). Another approach to co-create a visualization of the instructional design is to use a graphical educational modeling language, such as CoDe-Graph, which we will introduce below.

### Phase Three: Presentation and Documentation

The final phase of presentation and documentation brings the small groups back together to look at and discuss the resulting designs. Once the groups converge on their chosen model, they present it to the others in phase three. A graphical representation of the course is used to compare the design ideas from the small groups and discuss any discrepancies between the initial vision and the models. The facilitators and participants again ask questions, provide feedback, and eventually converge on a single design, which incorporates elements of the individual group designs. This acts as an evaluative step, letting the participants check if they have met the goals and requirements, they set for themselves in stage one. If alterations are needed, they can be implemented at this stage in an abbreviated design round focusing on the identified elements. During this process, the researchers document, both visually and in text form, the design decisions that are agreed on by the group. At the end of stage three, an initial design has been created, agreed upon, and translated into a graphical format, which can be clearly interpreted by all parties involved. This paves the way for rapid prototyping based on design documentation.

### Creating Shared Understanding Using Educational Modeling Languages

One approach to creating a common language among the design session participants supporting the design and presentation phases is to use an educational modeling language (EML). An EML is a way of representing a unit of learning. It is a tool to help orchestrate the different components of an instructional design, such as learning objects, activities, and assessments, and to show the learning path that students will follow to meet a learning goal. EMLs share some common characteristics as outlined by (Martinez-Ortiz et al., 2007). They are formally defined and machine readable, they are pedagogically neutral, be done, by whom and with what tools, and finally they should be resilient to technical changes such as switching platforms or tools.

One of the first modeling languages to be implemented was the EML-OU from the Open University of the Netherlands. It aimed to address the lack of pedagogical and instructional theories being implemented in Learning Management Systems (Martinez-Ortiz et al., 2007). Since then, numerous EMLs, and often accompanying software, have been developed and implemented in E-learning scenarios. These include the IMS Learning Design Specification (Koper & Olivier, 2004), participatory pattern languages (Mor & Winters, 2008; Mor et al., 2012) and the Integrated Learning Design Environment (ILDE; Hernández-Leo et al., 2018). Some EMLs look at formalizing collaborative learning environments (Weinberger et al., 2007), and in a few cases, EMLs have been based on business processing languages (Bergenthum et al., 2012).

Despite the variety of EMLs in use, most are either limited in their scope, showing a single learning process, only applicable in specified domains, or they are complex and require a large investment in time to understand and utilize the EML (Koper & Tattersall, 2005; Retbi et al., 2012). There is no such thing as the perfect EML which fits the needs of every design group, but still, one thing all the aforementioned EMLs do is to facilitate communication between technical and non-technical instructional designers (Martinez-Ortiz et al., 2007). Thus, EMLs have become one of the artifacts that have been used to solicit feedback and reactions from co-design participants (Matuk et al., 2016). EMLs and their graphical representations are used in design sessions as they allow participants to visualize the results of a discussion and clarify misunderstandings or miscommunications. In addition to being used for the final visualization, EMLs can be implemented as a tool to assist in the idea generation and exploration stage of designing. Hence, EMLs could be used to visualize courses on both the macro and micro-levels and could be accessible to all participants independent of technical knowledge.

### Description of the Language

Within the Competen-SEA project, which used co-design to develop MOOCs in non-traditional settings, the authors developed a graphical template (CoDe-Graph), to facilitate the exchange of design ideas. CoDe-Graph is based largely on the ideas of orchestration graphs and scripts, which combine different learning arrangements with each other (Dillenbourg, 2015; Kollar & Fischer, 2008; Kolling et al., 2019). In orchestration graphs, activities take place on one of three social planes, individual, team, and whole class. Edges, or connectors, show the relationships between activities (Dillenbourg, 2015). Håklev and colleagues (2017) adapt such orchestration graphs for use within MOOCs. Here, operators play a particularly key role as they automate many of the tasks traditionally done by the instructors (e.g., group formation or evaluating responses). Building on these ideas, we sought to create an adaptation of an orchestration graph that contained the basic elements of machine readable EMLs but would be highly accessible to non-experts while still providing pedagogical flexibility.

**Fig. 1:**
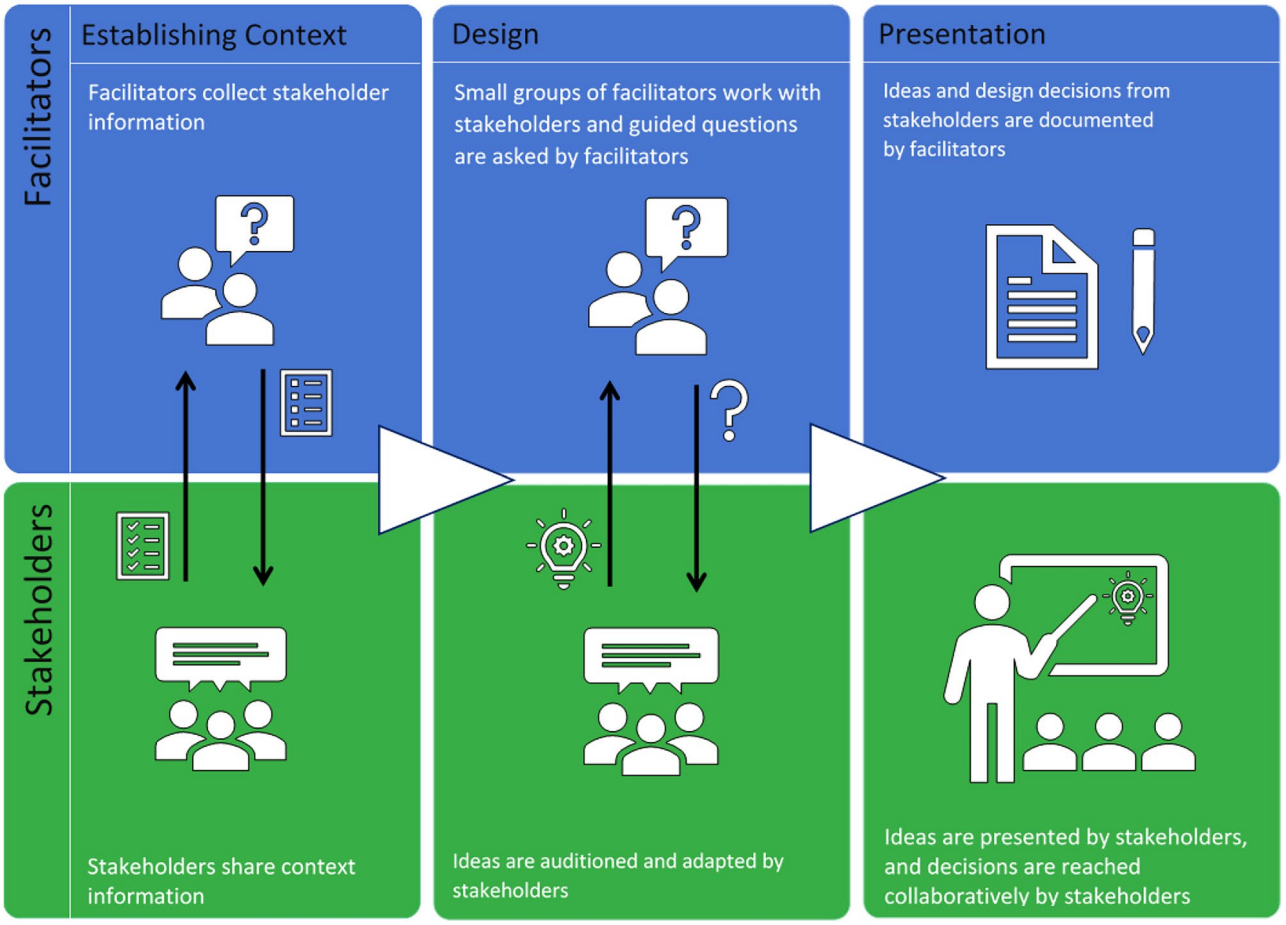
Co-design process model

**Fig. 2:**
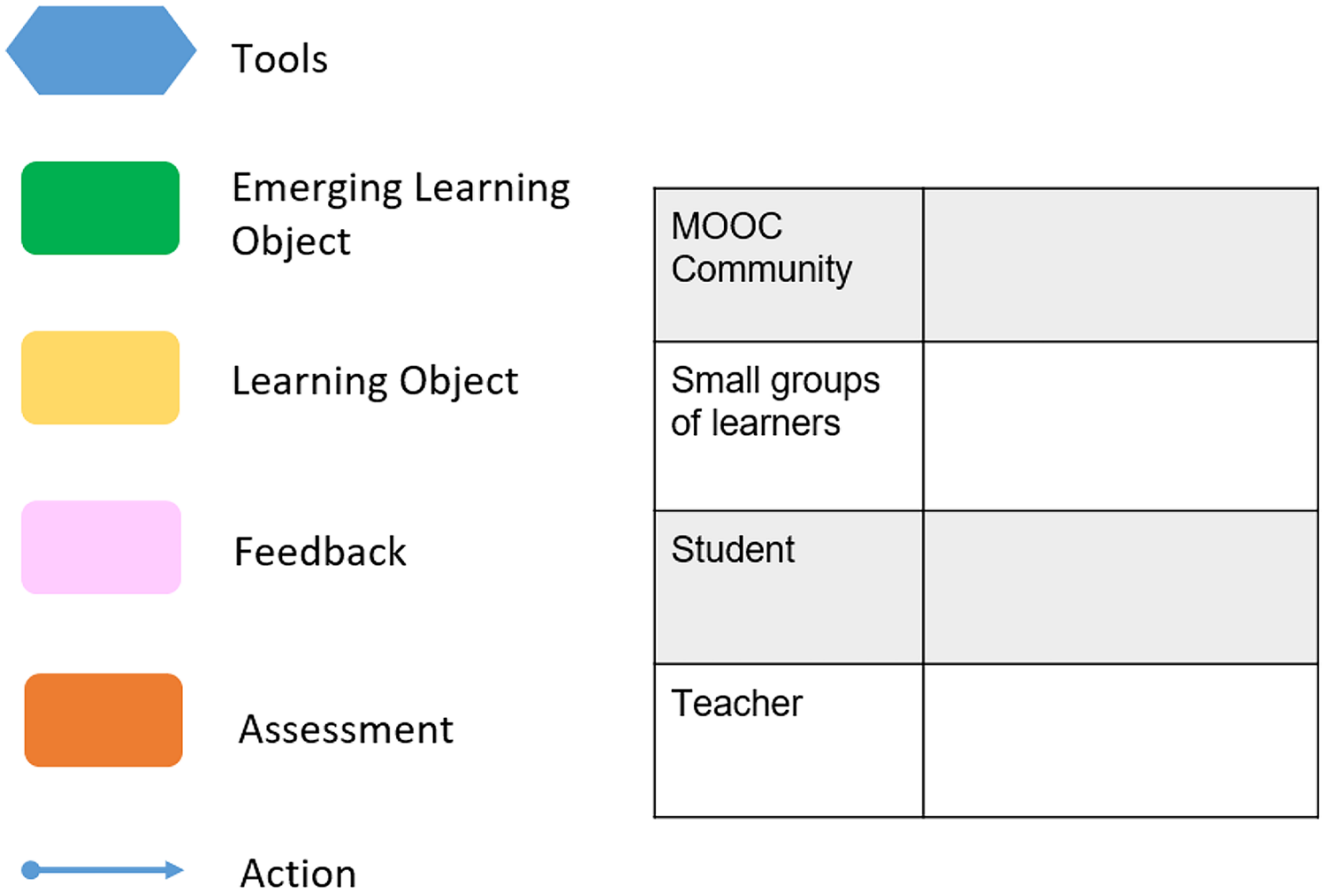
Building blocks of the CoDe-Graph template

CoDe-Graph templates (Figs. 1 and 2) can be used to show the flow of an entire course, a single unit, or a complex learning task. The X-axis represents the rough flow of time and the Y-axis the distinct levels of social interaction as identified by Dillenbourg (2015), with the addition of an instructor. Roles, as well as the learning resources, and activities are distributed across these levels. Course levels include (1) the “Teacher,” who can be an instructor, tutor, or even an automated system; (2) the “Student,” working individually; (3) the “Small groups of learners,” where students work together in groups ranging from randomly paired learners to cohorts, which share a start date, and (4) the “MOOC Community,” which represents all active participants in the MOOC. Five simple symbols, which can be annotated and labeled, are used to show activity within the MOOC. “Tools” indicate items such as forums, links to external resources, automated systems, and media. “Learning objects (LO)” are given artifacts; these often consist of learning resources such as videos, readings, audio, or images. In contrast, “Emerging learning objects (ELO),” student artifacts, include anything that is altered or created during course runtime, such as a quiz or a student-produced blog entry or graphic (Damșa, 2015; de Jong et al., 2010; Lejeune et al., 2009). These ELOs may in time become LOs for other students to use as a resource. Activities can be represented in a series of LOs and ELOs that interact with each other on varying social levels. There is also the option of representing “Feedback” from peers or the instructor. Finally, arrows are used to indicate actions, including, but not limited to, the production of LOs and ELOs, participation in course discussions, integration of feedback, and sharing of resources.

**Fig. 3:**
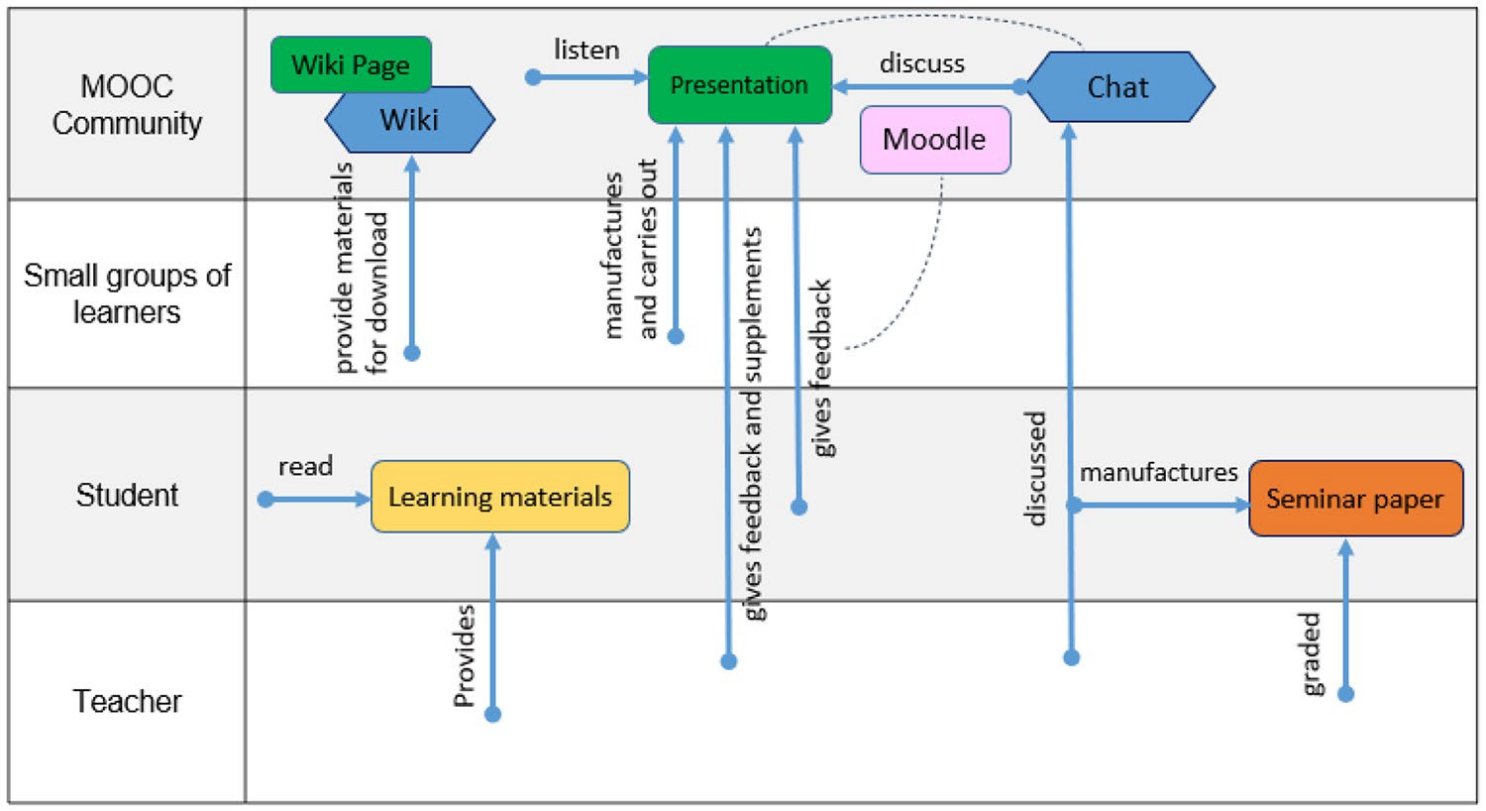
Instance of a seminar course designed with Co-De-Graph

An example of the CoDe-Graph language is presented below (Fig. 3). In this course, the instructor provides some materials for the students, while others are created by small groups and shared via Wiki. The students present their work to the whole class. Individual students can discuss the presentations via a chat function and then give feedback on the presentations via Moodle. Finally, individual students create and submit a term paper, which is graded by the instructor.

## Co-design in Practice

The co-design process model and graphical template, CoDe-Graph, were used in the Competen-SEA project (Competen-SEA.eu) to facilitate the designing of five MOOCs for underrepresented and disadvantaged populations. The Competen-SEA project was an EU capacity-building project, which established a partnership between European and South-East Asian universities. To this end, the European partners held workshops on MOOC design and implementation and the South-East Asian partners provided information about the local context and the curriculum. A co-design approach was deemed especially suited for this project, as no individual university had the needed expertise to create courses independently from the others and each strove to build their own skills and capacity through the project. Additionally, traditional MOOC designs were deemed insufficient for the types of highly specialized courses being offered and therefore, existing designs could not be replicated.

Each case was unique in content, goals, and challenges, a summary of which can be found in Table 1. It was clear that the traditional xMOOC, self-paced and reliant on lectures, articles, and quizzes would not be suited for most of these groups. Many participants lacked the skill sets needed to complete highly academic tasks independently and nearly all would be using instable technology to complete the courses. Under these circumstances, it seemed well advised to work with a wide range of experts to create suitable courses tailored to the target audiences’ needs.

**Table 1:** Commonalities and differences in MOOC case contexts

Disadvantaged groups	Single mothers (Malaysia)	A secluded community (Malaysia)	An alternative for fishermen (Indonesia)	Entrepreneurship (Indonesia)	Health workers (Philippines)
Course goals	Entrepreneurship, community building, empowerment, create role-models				
	Baking, beekeeping, and composting	Local knowledge and practices	Eco-tourism	Business and marketing plan	Data governance for health workers
Education	Mostly unemployed, partly school leavers or illiterate			Students and fishermen	12 years incl. health worker specialization
Challenges	Internet connection time limited, low, restricted to smartphones or community centers				
	Limited time- when children are sleeping	Computer literacy, dialect	Audio rather than video	Productive use of smartphones	

### Experiences During the Co-design Workshops

While the details of each co-design session varied slightly (e.g., by time, availability of technology, and number of participants), the frame of the sessions remained the same. Each group participated in a co-design session aimed at producing a design from which an initial prototype could be constructed. This session was structured around the three-phase co-design process model outlined above. The teams consisted of the two researchers leading the session, the local research team, content experts/teachers, and the technicians who would be leading implementation. Most participants had attended a 2-day workshop on designing and facilitating online classes in the months prior to the co-design session. Still, the level of expertise in areas such as design, pedagogy, and technical implementation varied among participants.

Each session began with introductions and goal setting and then moved directly into phase one—establishing context. The primary goal was to ensure that all participants had a similar understanding of the course context. This phase is tightly structured with facilitators working through a script of a pre-established series of questions in order to solicit information from the other team members. As expected, during this time, the stakeholders generally contributed the most turns (recorded transcripts from four cases indicate that in three of the four cases stakeholders contributed more than 60% of turns in stage one) and of the facilitator’s turns, many were of a procedural nature, i.e., asking the pre-established questions. In these cases, the stakeholders provided background knowledge about the subject matter and the targeted demographics. In addition, the first design requirements generally emerged within this discussion. For instance, the groups discussed the possible course and unit length based on the amount of time participants would likely have available to commit to the program. This allowed the group to establish some standards that could be used in the next phase of design, for example, in one case, it was decided that no single activity should take more than an hour, as the women participating would likely only be able to do so after their children were asleep. The information gathered in this phase formed the basis of the next-loosely structured phase, namely that of design.

**Fig. 4:**
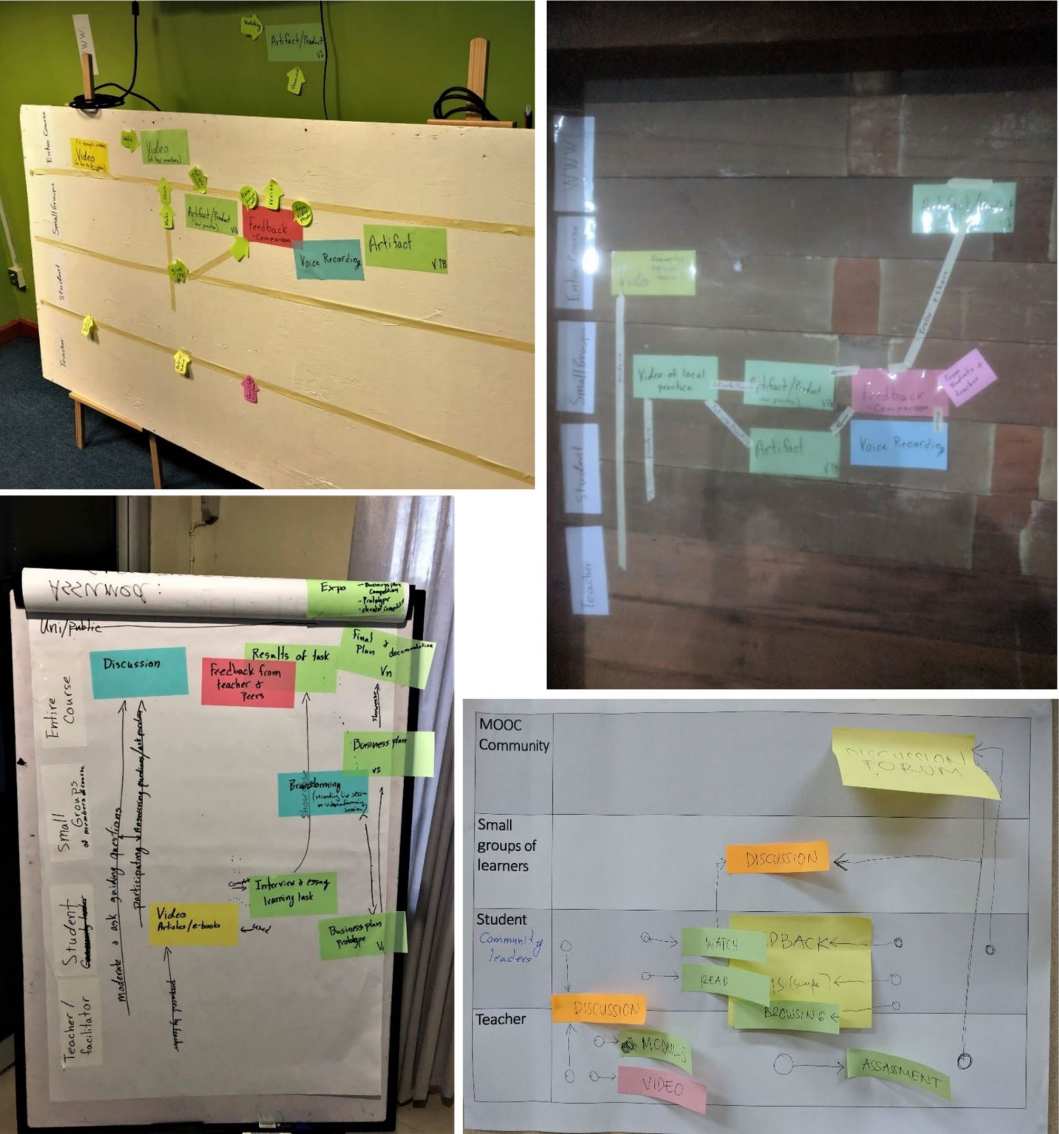
Examples of CoDe-Graph in use

**Fig. 5:**
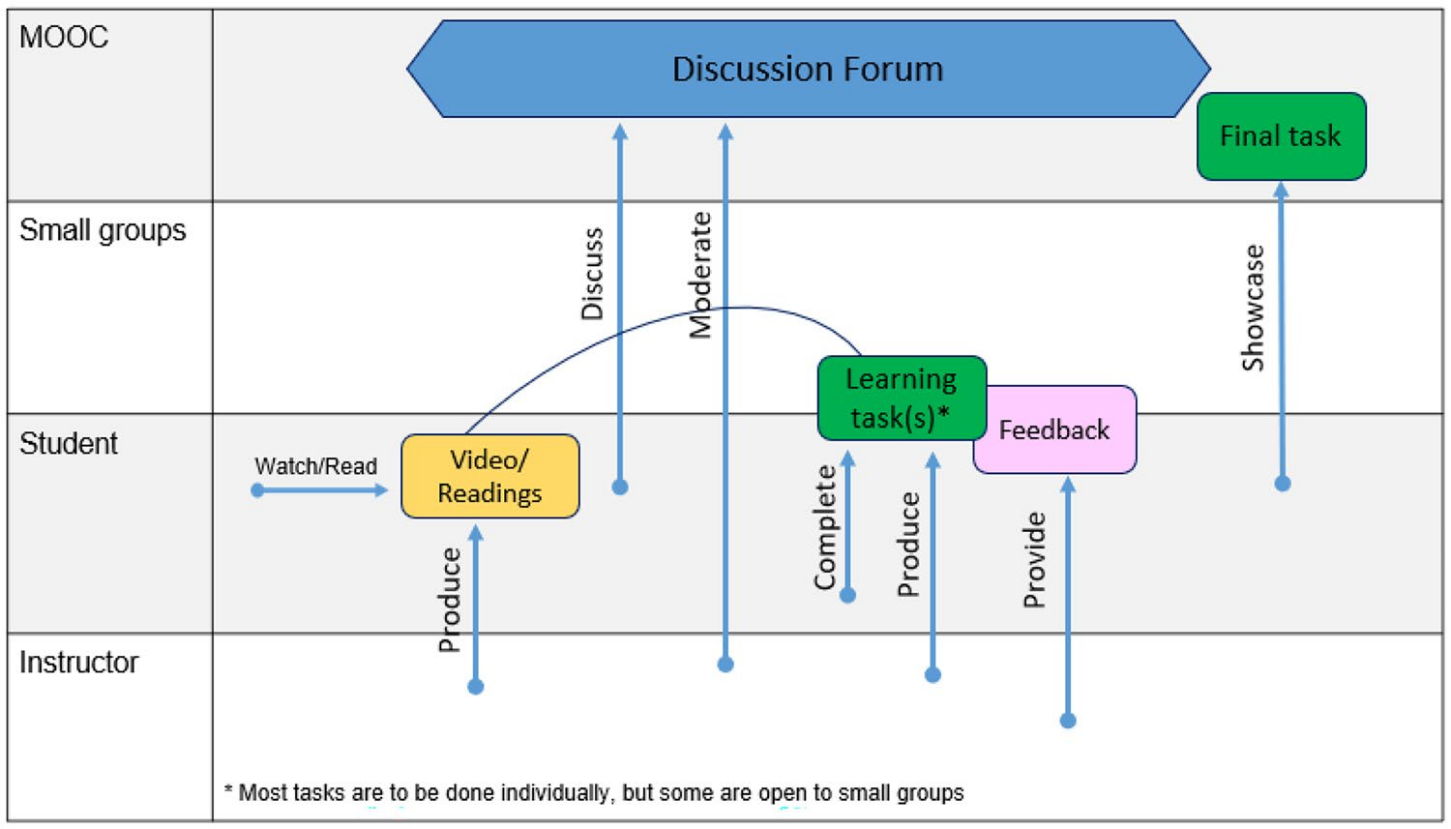
Design for “Single Mothers in Malaysia”

**Fig. 6:**
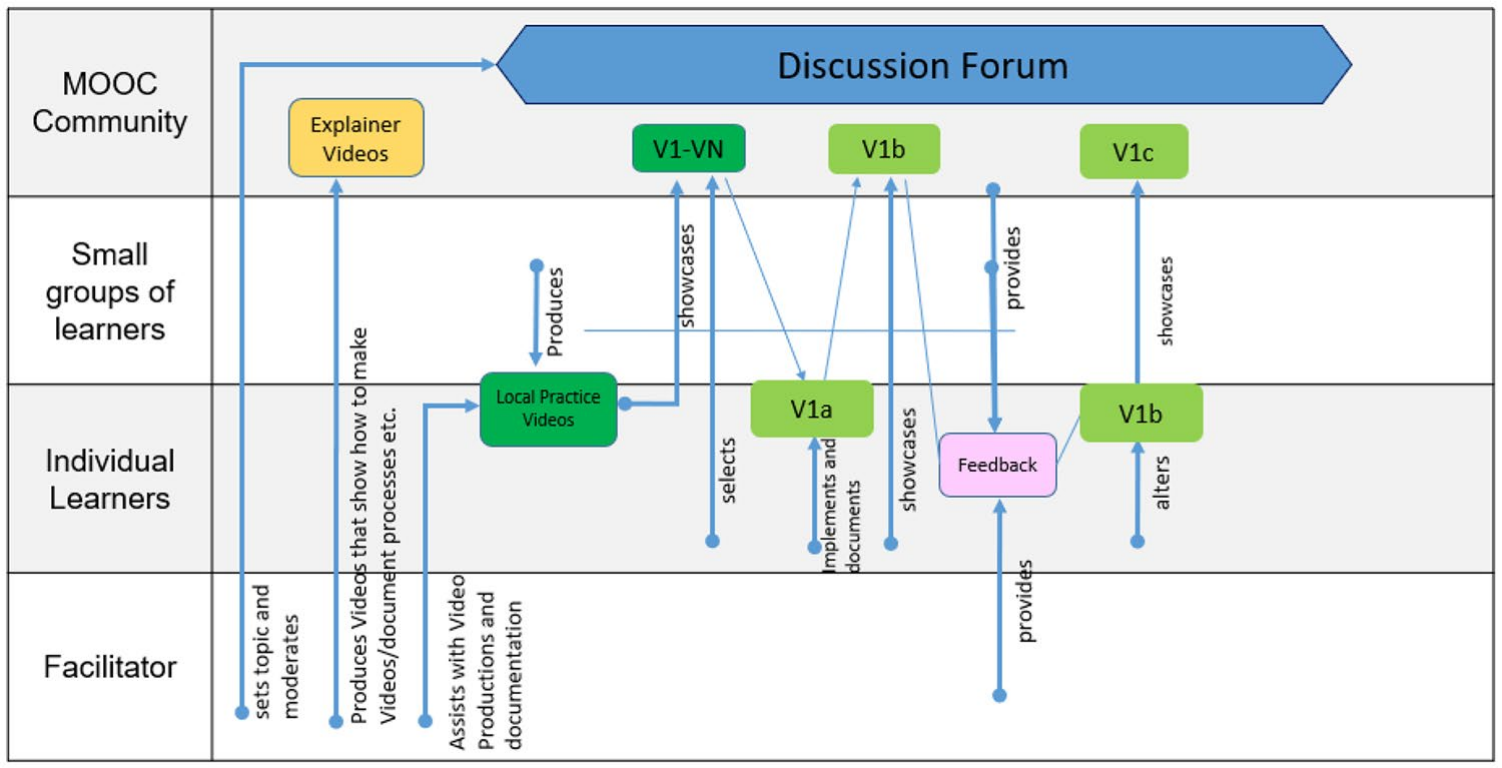
Design for “Secluded Community in Malaysia”

**Fig. 7:**
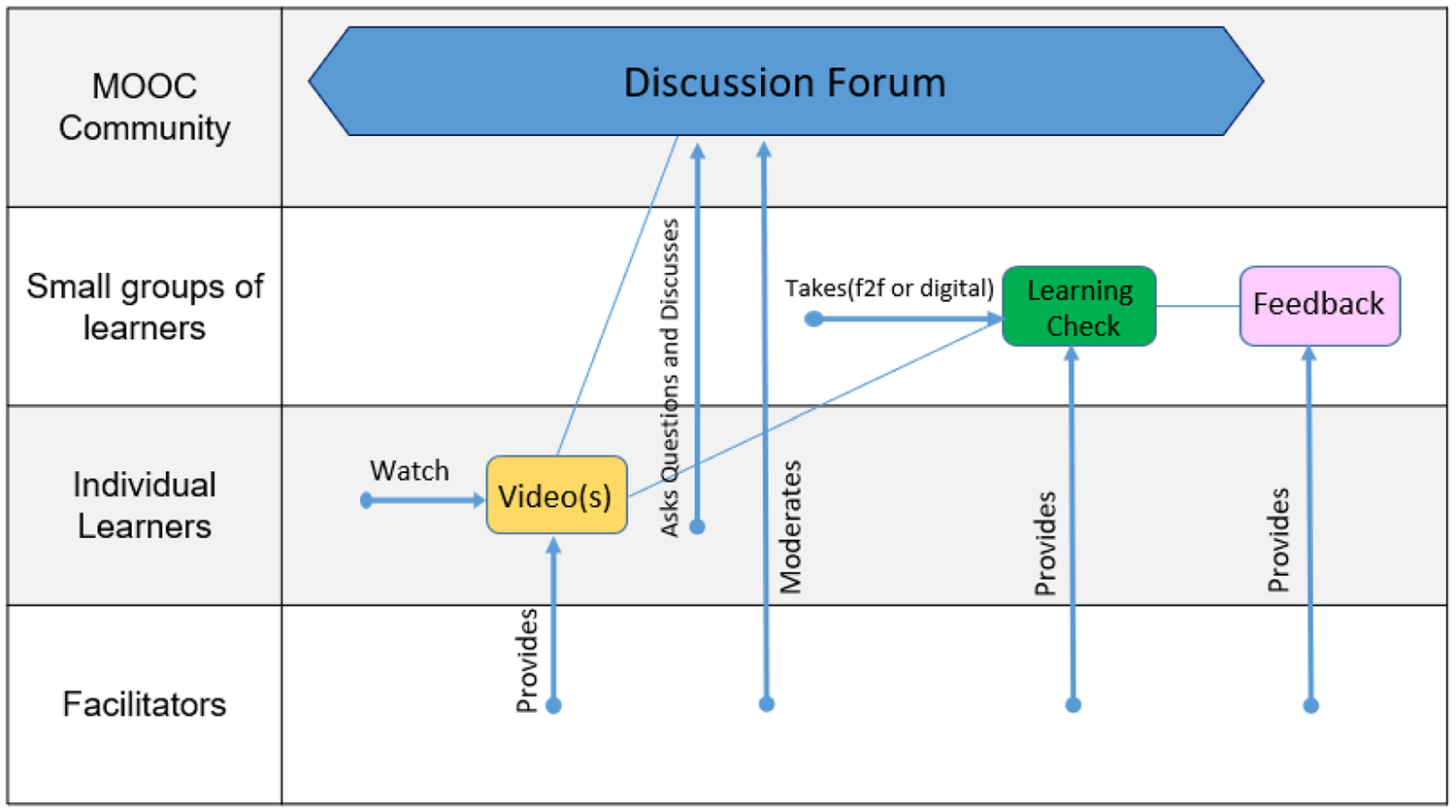
Design for “Alternative for Fishers in Indonesia”

**Fig. 8:**
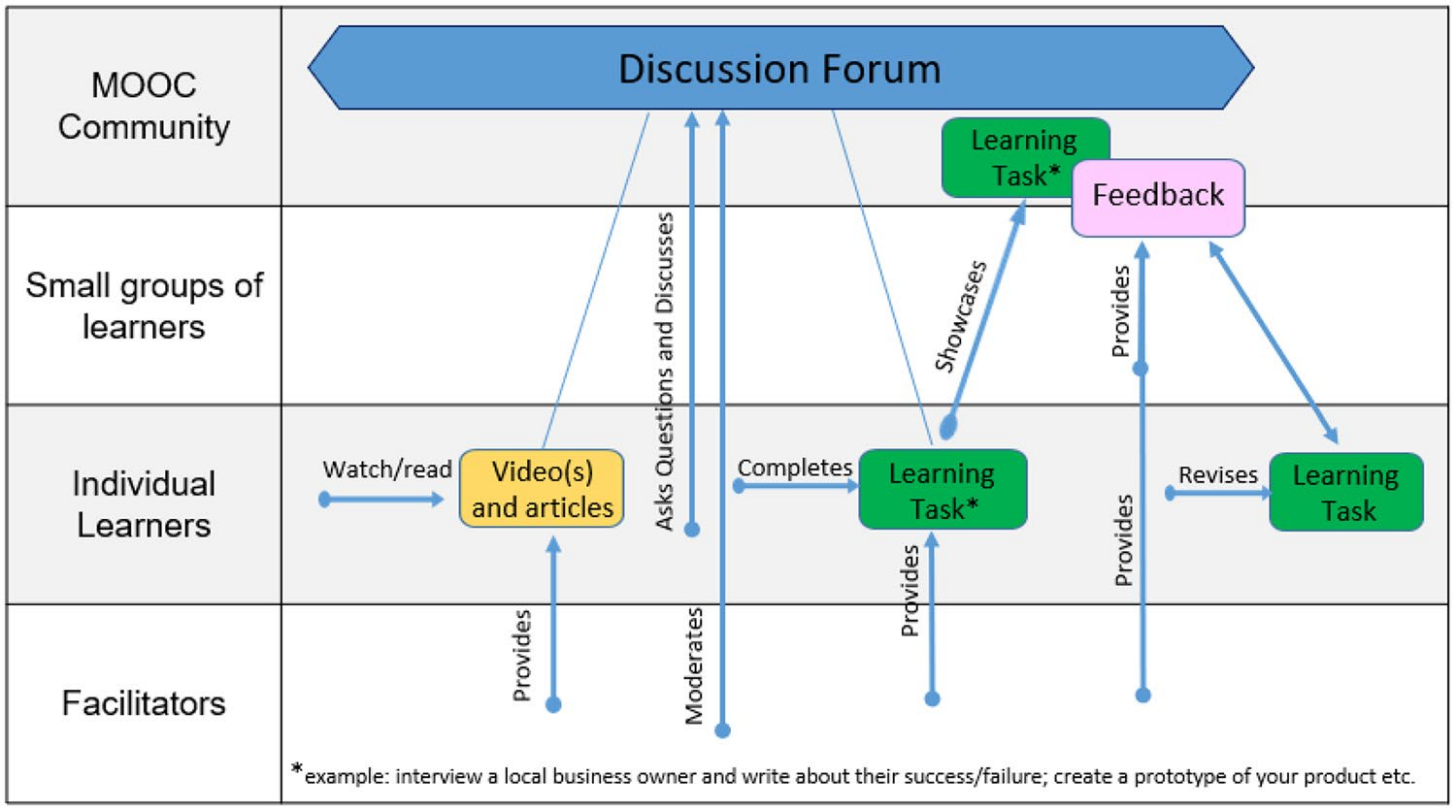
Design for “Enteprenuership in Indonesia”

**Fig. 9:**
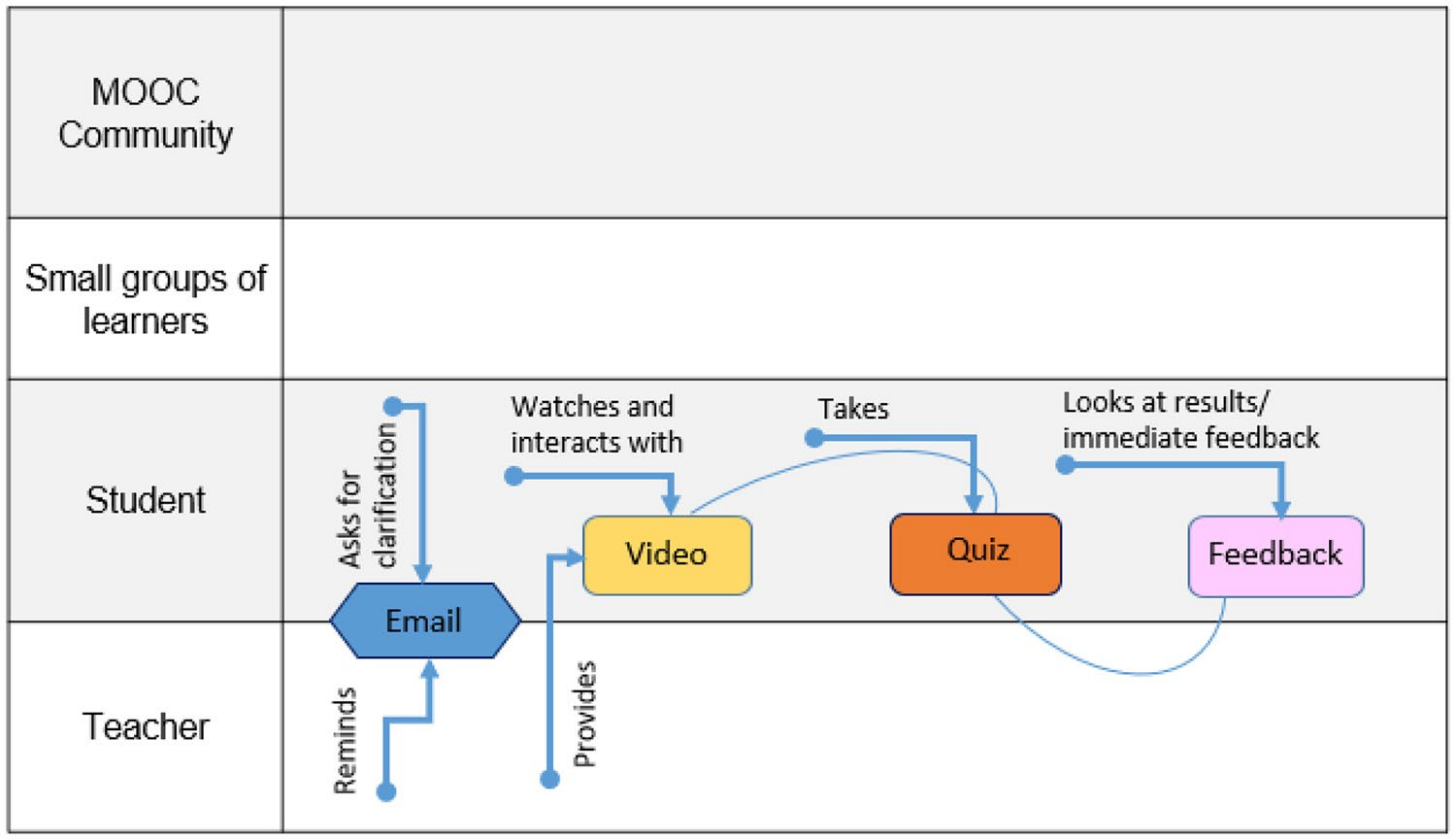
Design for “Rural Health Workers in the Philippines”

In phase two, large participant groups were split into smaller groups as needed and asked to work with the CoDe-Graph tool in order to create a draft model of at least one course unit. Due to the logistical challenges of the workshop locations an analog version of the CoDe-Graph template (seen digitally represented in Figs. 2 and 3) was used. Each group consisted of subject matter experts, pedagogical experts, a technician when possible, and at least one facilitator. In some cases, other stakeholders, such as an anthropologist, also took part. Participants were provided with large paper templates and color-coded note cards to use when creating their visual representations. The groups then worked together to audition ideas for the course. They physically shifted course elements around the template, adding and removing based on their groups’ feedback. As can be seen in Figs. 4, 5, 6, 7, 8, and 9, design drafts were created on pre-printed templates, flip charts, and walls depending on the available space and resources.

During this section, we see many instances of participants building on the initial ideas presented by their colleagues. For example, in one case, the idea of creating an individual video is proof of completion. This however evolved into having participant video their process not as an evaluation but rather as a resource for future students. Additionally, it went from an individual activity to a small group activity with many options for peer feedback. In addition, using the physical template helped members gain a shared understanding. In several cases, we saw groups correct their model based on discrepancies between members understandings. One participant would describe an activity, and another would place it on the model; however, in doing so, it became clear that there was a disconnection between the visions of the participants. Having a tangible model made this very clear and easy to identify in real time. This process of micro-iterations continued until each group was satisfied with the arrangement they had created.

In phase three, the groups conducted a formative self-evaluation, in which they reflected on their design and solicited feedback from the group. Each small group presented their initial design and the reasoning for their design decisions. The group then discussed to what extent it met the needs presented in phase one and what still needed to be improved. In cases where multiple groups submitted design, the group builds consensus as to which aspects of each design would be included in the final model. In a second, shorter iteration of the design, the workshop groups then refined the model based on the feedback.

The resulting designs were then translated into a standardized digital version and made available for the technical team to begin developing prototypes and the subject matter experts to begin creating learning materials. The resulting designs from each workshop are presented below along with a brief description:

### Single Mothers in Malaysia

The MOOC needs to be completed on a mobile device and accessible in micro-learning sessions to accommodate the busy schedules of working mothers. In addition, group work and forum participation are encouraged to grow community and create networks, which has been identified as a primary goal for the MOOC. This course consists of three units, all of which follow the same basic structure. Each unit consists of a theoretical and practical element. The facilitator provides information in the form of videos or readings and moderates the discussion forum throughout the course. During the theory sections, participants interact with the provided learning objects independently on mobile devices. The practical sections are completed independently, but participants can also opt to work in small groups. The goal of the MOOC was to help single mothers learn about attainable forms of entrepreneurship such as cooking, producing compost, or beekeeping. Group work may be particularly necessary where physical resources for single mothers to become independent, such as ovens, composting bins, and beehives, are not as readily available. The course ends in an in-person gathering where participants can showcase their work. Assessment occurs throughout the courses in the form of short closed-question quizzes designed to check for understanding. A final assessment of the created product is made at the end-of-course gathering. Additionally, participants will be assessed on their level of participation in the discussion forum and other online activities.

### Secluded Community in Malaysia

To share and sustain the local culture of a community secluded in the Borneo forests, participants in the course share their knowledge and expertise on local traditions and specialized regional knowledge (such as plant use, artist techniques, and folk tales). The course is highly informal, and participants use the setting to network and learn from each other. Members of the community are encouraged to spend about an hour of a week interacting with the content that is available and/or contribute new content to the platform. The platform acts as a repository for the knowledge created and shared. Participants have the opportunity to give feedback to each other and get support from the facilitators. On specific occasions throughout the year, participants will have the opportunity to showcase their knowledge at in-person events and receive recognition from the facilitators.

The course is set up for optimal flexibility. Facilitators assist in getting the learning network established and support participants as they navigate the learning environment. Facilitators provide in-person as well as video-based training about the platform and about producing content. Additionally, they moderate the discussion forum throughout the course. Participants view available material independently or in small groups (initial material will be produced by the facilitators) and to implement the skills, which have been presented. After trying out the material, they can post-digital documentation in the form of pictures, videos, or audio recordings. They can then leave feedback or add information for others who may want to try out the skill. Content can be created and shared in multiple formats, including video and audio for participants who may not be comfortable reading and writing. This creates a collection of experiences that members of the community can draw on. Participants also may submit their own original ideas and make them available for the rest of the community.

### Alternative for Fishers in Indonesia

In an overfished region, this course offers formal certification for area workers to engage in eco-tourism and sustainable fishing practices. Successful completion of the course opens additional sources of revenue for participants as well as helps them improve their current business models.

The course consists of five modules and takes approximately 30 h to complete. The course is a combination of online and offline activities that can be completed individually or in small groups. Small groups are encouraged due to a limited availability of tablets and also to allow participants to collaboratively build knowledge and promote discussion. Participants are scored on their participation and the results of group quizzes. However, to receive a full certificate of achievement, as opposed to participation, they must complete the final exam individually.

Each module consists of three units which have a similar structure throughout the course. The unit begins with participants watching a video that has been produced and provided by the expert/teacher. Individual participants then have the chance to pose questions and discuss their opinions in a forum. This forum is moderated by the teacher. Then, the teacher provides a learning check, primarily in the form of a closed-question quiz, which small groups of learners can work on together. Participants have the option of meeting in person to complete the quiz or they may interact within the digital space. The teacher then provides detailed feedback on the quiz.

### Entrepreneurship in Indonesia

This course aims to address a particularly wide breadth of potential participants including local business owners and university students. Due to this, the course is designed in a modular fashion so that participants can complete the entirety of the course or only the sections most relevant to their goals.

The course is based off an extensive semester-long in-person course but will be divided into smaller courses that can be taken separately and cover specific skills and knowledge. Students will primarily work individually but may have the option of completing some tasks in small groups. Students will give each other feedback and are expected to support one another. Formal assessments will take the form of an authentic learning task (e.g., giving an elevator pitch or creating a Facebook marketing campaign).

At the beginning of each section, the teacher provides a selection of learning objects for the participants to view/read/listen to and comment on in the discussion forum. Participants can also ask and answer questions in the moderated discussion forum. The teacher provides a learning task based on the earlier material. The participant completes the learning task and showcases the results in the MOOC community. The teacher and fellow participants then provide feedback, and the participant has the chance to make revisions and repeat the feedback cycle until they are satisfied and then digitally showcase their results.

### Rural Health Workers in the Philippines

The course is based off previous in-person training events and aims to make the training more widely accessible and efficient in terms of time and resources. In contrast to the other cases, the participants all have a secondary education and formal vocational training. While technology is somewhat more reliable and available in this case, most participants still have a low level of digital literacy. The design team also did not have the same level of flexibility in this case as many elements, such as learning resources and platform functionality were strictly regulated by governmental authorities.

The teachers provide animated videos on the topic. The participant watches the video and interacts with the provided material. They have the option of communicating with the teachers via email should they have questions or comments. Once they feel comfortable with the material, they complete the closed answer question quiz and receive instant automated feedback. They can then proceed to the next unit.

## Discussion

This initial work in co-designing MOOCs for targets populations should serve as a model both when considering what customized MOOCs may look like, and as a possible guide to working in highly diverse co-design teams. Taken together, the examples above provide some first pointers towards how co-design can lead to different MOOC designs ranging from x- to cMOOC formats, which are responsive to the specific, highly varied contexts of disadvantaged groups of learners. By utilizing the wide range of expertise, experience, and skills in a team, co-design can create unique custom MOOCs that are adapted to the specific needs of its target populations. However, co-design is more than a group of people sitting around a table and brainstorming ideas. Facilitation is needed to guide the process towards implementable designs and prototypes (Penuel et al., 2007). In addition, tools such as shared representations in EMLs, ease the co-design process by giving participants a common language to use when discussing the course vision and design (Barbera et al., 2017). While being strongly guided through a specified process of co-design, the respective stakeholders in different regions of South-East Asia have created these designs rather than taking over one-size-fits-all MOOC designs.

Conceptualizing co-design as a facilitated process a team moves through iteratively allows to separate structure and facilitate each stage to meet the group’s needs. The co-design teams in our study reached intermediate targets with every completed phase, which gradually moved the group towards the finished design. In each case, the initial design was created within a single day from which educators and technicians could build on and continue refining and prototyping over the course of the next weeks.

In addition, following the process model provides chances in each phase to correct the misunderstanding, a common pitfall in co-design (Penuel et al., 2007), and create a shared vision. The use of structured processes and languages, such as CoDe-Graph to produce co-designs, can contribute to the creation of a shared understanding and the generation of design ideas. Establishing context through a set of guiding questions provides participants the chance to collaboratively construct a common base of knowledge around the context, challenges, and goals of a course. Thus, misunderstandings can be immediately identified and corrected before the group even begins designing.

Similarly, using an EML or graphical template allows participants to clearly express their ideas and propose adaptations and alternatives. Co-creating a graphical representation of the instructional design enables stakeholders to discuss and work on the design before it is being implemented and in the case of an instructional design remains intangible even when it is implemented. Moreover, participants can create many versions of a single idea, auditioning possibilities until the group builds consensus as to the best possible solution.

This initial work in co-designing MOOCs for targets populations should serve as a model both when considering what customized MOOCs may look like and as a guide to working in highly diverse co-design teams.

## Limitations

We would like to point out some limitations and points of discussions of CoDe-Graph. The graphical template was designed to be able to accommodate a variety of course formats and indeed the co-design processes resulted in a wide breadth of instructional designs. While the graphical template could be applied in versatile ways, it is not pedagogically neutral. The mere presence of social levels intentionally encourages consideration of combining different learning arrangements. In addition, the distinction between LO and ELOs seemed to have a similar effect with groups brainstorming possible ELOs that would fit their context. In this way, CoDe-Graph reflects the ambivalence of co-design of both, involving stakeholders to freely create and design instruction, and at the same time providing some level of guidance to scaffold the process—here, to suggest design elements and patterns through the components of the language. Nevertheless, the designs, and eventually resulting courses, were highly individual and tailored to the communities they served. So, the language can express a variety of designs. Noticeably, the emerging designs line up on a dimension between x- and cMOOC and it might be interesting to consider more qualities and types of designs of MOOCs to understand how CoDe-Graph would be able to express multiple design types.

Last, but not least, CoDe-Graph’s main feature is its simplicity, which allows it to be learned and applied instantly. This in turn comes with simplifications and caveats in comparison to other attempts of graphical design languages that would encompass and define any conceivable feature of the learning design and allow easy, i.e., direct coding for a technical learning platform (e.g., Dillenbourg, 2015). Still, CoDe-Graph remains versatile and simple to understand, while being non-ambiguous.

## Future Research

The process model of co-design and CoDe-Graph was developed in the context of a European project facilitating the design and development of MOOCs for disadvantaged groups in South-East Asia, a context that particularly affords co-design for productively combining different types of insights and expertise. Both, the process model and CoDe-Graph have ever since been applied in multiple projects as well as COVID-19 emergency online learning design sessions to help develop different formats of online courses.

The high potential may lay in systematically combining the co-design approach with existing, formative approaches of instructional design in the future, such as SAM. This could lead to categorizing future approaches along the dimensions of how a design process is structured in time, including how the design process is iterative and reactive to assessment, and how a design process is distributed over different stakeholders, including who is feeding back information on the design’s successes and shortcomings. Specific value could lie in expanding co-design over multiple iterations. Future iterations of designs could then be informed by an enlarged group of stakeholders, including past and future students in an evaluation and re-design phase.

The next phase of our research will focus on improving our understanding of how these tools impact co-design interactions. Additionally, we seek to identify characteristics of design groups and sessions, which may impact the effectiveness of facilitating co-design teams with CoDe-Graph or similar design tools. Future research is also needed to investigate the relationship between levels of guidance and degrees of freedom in co-design, the dependencies of how domain, context, and design knowledge interact in processes of co-design, as well as on how co-design can realize goals of sustainable, accepted, and effective designs for learning.

## Data Availability

Transcripts of design sessions are available upon request.
